# Children’s pre-academic school readiness and home learning activities: a moderated-mediation analysis of home visiting

**DOI:** 10.3389/fpsyg.2023.1245893

**Published:** 2023-10-26

**Authors:** Daniela Avelar, Darrell Hull, Wendy Middlemiss

**Affiliations:** College of Education, University of North Texas, Denton, TX, United States

**Keywords:** school readiness, home visiting, moderated mediation, prekindergarten, home learning environment, early childhood, parent engagement, home learning activities

## Abstract

**Introduction:**

The current study explores the relation between parent involvement and children’s school readiness for 568 families enrolled in the Home Instruction of Preschool Youngsters (HIPPY) program in Texas. Parent involvement in children’s learning is a focus of the HIPPY curriculum.

**Methods:**

In this analysis, conditional process models were run to examine the relations between children’s school readiness and engagement in home learning activities, parents’ education level, program language (English or Spanish), both before and after completing a year of the program.

**Results:**

At pretest but not posttest, program language moderated the direct relation between parents’ education level and children’s school readiness and parents with high levels of education were more likely to engage their children in educational activities. Engagement in home learning activities was associated with school readiness regardless of parents’ education level and language.

**Discussion:**

These findings provide a better understanding of the variables associated with school readiness for HIPPY families, indicating the importance of home learning activities—a variable that may be more amenable to change within intervention programs. Thus, focusing on home learning activities may contribute to decreasing discrepancies in children’s preparedness for school entry that are generally identified across language and parent education. Findings may have practical implications for other home visitor programs working with similar populations.

## 1. Introduction

Learning is a social process through which children acquire knowledge, skills, and an understanding of cultural practices (Vygotsky and Cole, 1978). Their social interactions with caregivers support thought and language development, contributing to development of self-regulative capacities and academic preparedness (Wertsch, 1979; Taber, 2020; Smolucha and Smolucha, 2021). As supported by theory of cognitive development, children’s guided interactions and collaborative dialogs with more knowledgeable or skilled adults, provide opportunities to scaffold learning through simplified language, modeling, visuals, cooperative learning, and hands-on learning (Ovando et al., 2003).

The frequency and quality of parents’ scaffolding of children’s learning and language development provides children opportunity for practicing their language skills (Hart and Risley, 1995; Camp et al., 2010). While children will develop language naturally, parent–child interactions can create a richer home learning environment and facilitate children’s memory, attention, and concept formation across the four stages of language development (Gredler, 2009). Although stages are not necessarily incremental or discontinuous in nature, they represent benchmarks of cognitive developmental progression. With continued interaction and scaffolded experience children move from a primitive/natural stage of behavior where pre-intellectual speech is intertwined with pre-verbal thinking (Wertsch and Sohmer, 1995) to the defining of how tools can be used and form the first operations of a practical mind. With continued development, children internalize mental operations that help form behavior, eventually moving through stages marked by for solving internal mental tasks based on external signs, such as counting on fingers or use of mnemonics for remembering. Finally, with continued support children move external operations to an internal mental plane, evidenced by completely internal speech that guides thought, such that memory is based on internal relationships to produce logic, or more overtly, in mathematical operations counting occurs completely internally. This is the genesis of internal speech that is prominent in adult thought and language use (Vygotsky and Cole, 1978).

Home visiting interventions can teach parents how to engage their children in language and learning activities that will strengthen children’s skills and help them succeed in school. Moreover, home visiting programs can help create equal access and opportunity for children at risk of low levels of school readiness by working with families in an environment in which they are comfortable (Sweet and Appelbaum, 2004; Astuto and Allen, 2009). Children who acquire pre-academic skills in their preschool years have a greater likelihood of successful entry to school, which is also associated with higher academic achievement later in school, graduation from high school, and securing employment (e.g., Duncan et al., 2007; Claessens et al., 2009).

### 1.1. Early childhood program access

Meta-analyses indicate home visiting programs enhance the quality of home learning environments and support parent–child interactions (Kendrick et al., 2000; Nievar et al., 2011; Filene et al., 2013) and children’s cognitive outcomes (Comfort, 2003; Sweet and Appelbaum, 2004; Filene et al., 2013). Home visiting programs remove barriers to attending center-based such as transportation, financial struggles, no childcare, and work or scheduling conflict (Peacock et al., 2013). Additionally, in a home visiting setting, home visitors observe families in their own environment and learn more about their family dynamics, establish rapport, and provide undivided and personalized attention (Sweet and Appelbaum, 2004), elements of service that can support program engagement.

Although many home visiting programs are successful, in the United States there remains a gap in early school preparedness based on socioeconomic resources and primary language (Janus and Duku, 2007; Peterson et al., 2018). Home visiting programs should find ways to equitably support children who may face risk factors for school entry based on ecological factors and identify successful program components to support children’s school readiness.

Thus, the current study explores how one home visiting program encourages parental involvement in culturally and linguistically diverse families. We explored parent–child interactions as related to children’s school readiness, while examining the moderation factors of parents’ education level and primary language (i.e., factors often associated with children’s school preparedness) (Becker, 2011). Focusing on these aspects of children’s cultural context, we investigate if parent involvement mediates the interaction between parents’ education and school readiness and if language moderates the effect of education on school readiness of children enrolled in HIPPY, a well-established home visiting program. Second, we examine if these relations change before and after the HIPPY program year.

#### 1.1.1. HIPPY program

As a home visiting program focused on working with parents to enhance parent–child interactions around educational activities, the Home Instruction for Parents of Preschool Youngsters (HIPPY) program can foster children’s progress through cognitive benchmarks and foster school readiness. Acquiring pre-academic reading and math skills starts at home and contributes to children’s school readiness (Daily et al., 2011; Sabol and Pianta, 2017). School readiness includes physical wellbeing and motor, socioemotional, language, and cognitive development which have been shown to promote positive learning and classroom expectations (Emig, 2001; Mashburn and Pianta, 2006; Duncan et al., 2007).

The HIPPY program is an empirically based home visiting program created in Israel in 1969 (Lombard, 1981) and adapted to operate in 15 countries in seven languages. The program curriculum focuses on helping parents of preschoolers gain confidence in providing their children early scaffolded learning experiences. HIPPY includes a 30-week curriculum during which home visitors role play with parents as a means of teaching parents how to engage their children in interactive learning activities to strengthen language, literacy, science, math, and motor skills. By increasing their involvement with their children’s education, parents can prepare their children for school.

The effects of HIPPY for both parents and children have been studied extensively. A meta-analysis revealed that the overall effect of HIPPY on children’s academic and behavioral outcomes was 0.48 (Goldstein, 2017), representing a moderate effect. HIPPY helps children achieve school readiness at kindergarten entry (e.g., Brown and Johnson, 2014; Brown and Lee, 2017; Abdulaziz, 2022). Specifically, HIPPY enrolled children were almost two times more likely to pass school readiness assessments in kindergarten than their non-HIPPY peers (Payne et al., 2020). HIPPY children also outperform their non-HIPPY peers in math and reading state mandated assessments at multiple grade levels (Brown and Lee, 2017; Abdulaziz, 2022). Program participation supported HIPPY parents’ involvement with their children’s learning (Nievar et al., 2011; Johnson et al., 2012; Brown and Johnson, 2014; Nathans et al., 2020). HIPPY program mothers reported higher levels of parenting self-efficacy, confidence as their children’s teachers, more learning materials at home, and engaged in more learning activities than mothers on the waiting list (Nievar et al., 2011).

HIPPY home visitors are members of the same community and have similar cultural backgrounds as HIPPY families and most were former HIPPY parents. In line with sociocultural theory, home visitors are the more knowledgeable peers who role-play with parents thereby scaffolding different learning activities every week. Parents acquire the skills to engage with their children and guide them through the school-readiness related activities, providing the appropriate support. For instance, for shared reading, home visitors model the types of questions to ask the child during reading to promote engagement and comprehension. In relation to reading, for example, parents learn and practice the role-played activities and then apply them when reading to their child, thereby scaffolding the child’s reading through the shared and helping children make meaning from texts by asking their child increasingly more challenging questions about the book (e.g., Whitehurst et al., 1988). Reading becomes a collaborative learning experience, first between the home visitor and the parent, and then between the parent and the child. A central sociocultural element of the program is the focus on parent involvement in activities with their children. Even though multiple studies have evaluated HIPPY’s impact, there is limited research on how parents’ involvement and factors such parents’ level of education and language preference may influence the program’s effectiveness. The goal of this study was to obtain a better understanding of the role of parent involvement on children’s school readiness for families enrolled in HIPPY.

### 1.2. Potential factors associated with school success

#### 1.2.1. Socioeconomic status

Numerous studies have found family SES (income, parents’ education, and/or employment status) is related to children’s school readiness (e.g., Duncan et al., 1998; Yeung et al., 2002; Janus and Duku, 2007; Piña et al., 2020). Children from moderate to high-income families tend to score higher in school readiness assessments than peers from families with fewer resources. Low SES families tend to face hardships such as higher levels of stress, reduced access to educational resources and materials, unstable work schedules, and financial constraints (Barnett and Hustedt, 2005) that may prevent them from engaging in educational activities with their children, which can in turn impact children’s success in school. Additionally, low SES families tend to have less knowledge of child development (Rowe et al., 2016), which is important to foster a home learning environment with cognitively stimulating activities (Vernon-Feagans et al., 2008).

Children whose mothers have higher levels of education are more likely to perform better in reading, math, and general knowledge assessments (e.g., West et al., 2000). Parents’ education is associated with the provision of developmentally appropriate learning and educational materials and availability and access to resources. A recent study found that 49% of 3- to 5-year-old children with parents possessing a high school diploma or less were rated as “on track” for early learning skills, compared to 66% of children whose parents have a bachelor’s degree or higher (Ghandour et al., 2021).

#### 1.2.2. Home learning environment

One of the factors through which SES and school readiness are linked is the home learning environment, the ways in which parents support their child’s learning by providing learning materials and engaging them in home learning activities (HLA) that support their cognitive development. A positive home learning environment can increase children’s school readiness by developing pre-academic skills thereby having a positive impact on children’s achievement later in school (e.g., Fantuzzo et al., 2004; Downer and Pianta, 2006). Parents who engage their children in language and literacy interactions, encourage children to ask questions, provide multiple learning opportunities in and outside the home, and partake in shared reading interactions, are more likely to have children with higher academic achievement at school entry (Edwards et al., 2008; Puccioni, 2018; Paschall et al., 2020). Furthermore, positive change in the quality of home environments throughout children’s preschool years was associated with greater gains in children’s school readiness skills (Korucu and Schmitt, 2020).

Home learning environments vary by family demographic characteristics, such as income, parents’ level of education, race, and language proficiency. Numerous studies have found negative effects of poverty on children’s home environments and on parent–child interactions (e.g., Huston et al., 1994; Evans, 2004; Fantuzzo et al., 2004). Parents who have a lower level of education, on average, spend less time engaging their children in home learning activities than parents from mid-SES backgrounds (e.g., Vandermaas-Peeler et al., 2009). Furthermore, a rich home learning environment seems to mediate the relation between SES and school readiness. For instance, Yeung et al. (2002) found that providing an environment that stimulates children’s learning mediated the relation between income and 3- to 5-year-old children’s cognitive achievement scores in letter-word and applied problems assessments. Others have found differences in this mediation based on race/ethnicity. For instance, Dotterer et al. (2012) found that sensitive parent–child interactions mediated the relation between SES and school readiness for White families, but not for African American families. Negative/intrusive parenting was a significant mediator for both groups, indicating that a decrease in SES was associated with more negative/intrusive behaviors, which were related to lower children’s school readiness scores.

Few studies have examined the relation between family demographics, HLA, and school readiness within the context of interventions designed to strengthen parental involvement. Of those that have been reported, one study found that higher SES was associated with more time spent doing activities at home, which in turn was associated with more gains in math and literacy school readiness skills for families enrolled in an intervention to support school readiness skills (Marti et al., 2018). Furthermore, children’s whose parents had at least completed high school scored higher on language assessments and English-dominant children scored higher on early math and literacy assessments than Spanish-dominant children. Similar findings have been found for children enrolled in Head Start (Parker et al., 1999). Enhanced parent–child relationships, home learning environments, and parents’ understanding of play from pretest to post-test were related to higher school readiness. The current study expands on these findings by examining the role of HLA on children’s school readiness scores before and after completing a year in the HIPPY program.

#### 1.2.3. Language, ethnicity, and immigration status

Speaking Spanish is not a risk in supportive environments, but limited English proficiency combined with limited access to physical and educational resources presents clear barriers to helping children become successful in school. Hispanic families have strengths since they highly value their children’s education and have high educational expectations and aspirations for their children (e.g., Ryan et al., 2010). However, Hispanic children in the United States, particularly those with parents who are immigrants, are more likely to be living in poverty, have parents with lower education attainments, have fewer resources, and are less likely to be fluent in English than other immigrant or native-born Hispanic parents (Aud et al., 2013; López and Foster, 2021). At the start of kindergarten, there are significant gaps in reading and math between Hispanic children and their non-Hispanic White American peers (Reardon and Galindo, 2009). Specifically, first- and second-generation immigrants from Mexico and Central America and children from Spanish speaking homes have the lowest math and reading scores when they enter kindergarten (Reardon and Galindo, 2009). Among children with immigrant parents, children with English-speaking parents score higher in school readiness assessments than children with non-English speaking parents (Lahaie, 2008). A National Survey of Children’s Health revealed that 54% of 3- to 5-year-old children from home where English is the primary language were considered healthy and ready to learn compared to 38% of children from homes with a language other than English, but these differences were no longer significant after controlling for socio-demographic factors (Piña et al., 2020).

The language of school readiness assessments should also be considered. At the end of preschool, Latino dual language learners performed as well as their monolingual English-speaking peers on school readiness (López and Foster, 2021). Specifically, Spanish dominant children scored well on assessments in Spanish, but below the norms in English (López and Foster, 2021) highlighting why it is important to assess children in their dominant language or in both languages to fully capture their knowledge. Yet, assessments are typically administered in English since it is the language of instruction in schools in the United States. In the present study, we assessed pre-academic school readiness skills in the family’s primary language.

Home visiting programs can provide resources and information to foster parent–child interaction and skills to foster children’s cognitive development. Giving families the option to receive the program in English and Spanish is culturally responsible, encourages participation, and makes families more likely to benefit from the program. Using parents’ preferred language can also make parents feel more comfortable and confident to complete the program. Because HIPPY focuses on parent–child interactions and home learning activities parents need to understand the activities and skills and be able to apply them so giving them the choice to complete the program in their preferred language can facilitate high quality interactions.

Because language status alone does not place children at risk for not being ready for school (López and Foster, 2021), the current study accounts for the interaction between language and parents’ education by using language as a moderator in the relation between parents’ education and home learning activities and parents’ education and children’s school readiness. Additionally, it evaluated if families receiving the program in English or Spanish are benefitting equitably by examining if language differences are present at posttest.

### 1.3. Current study

**Figure 1:**
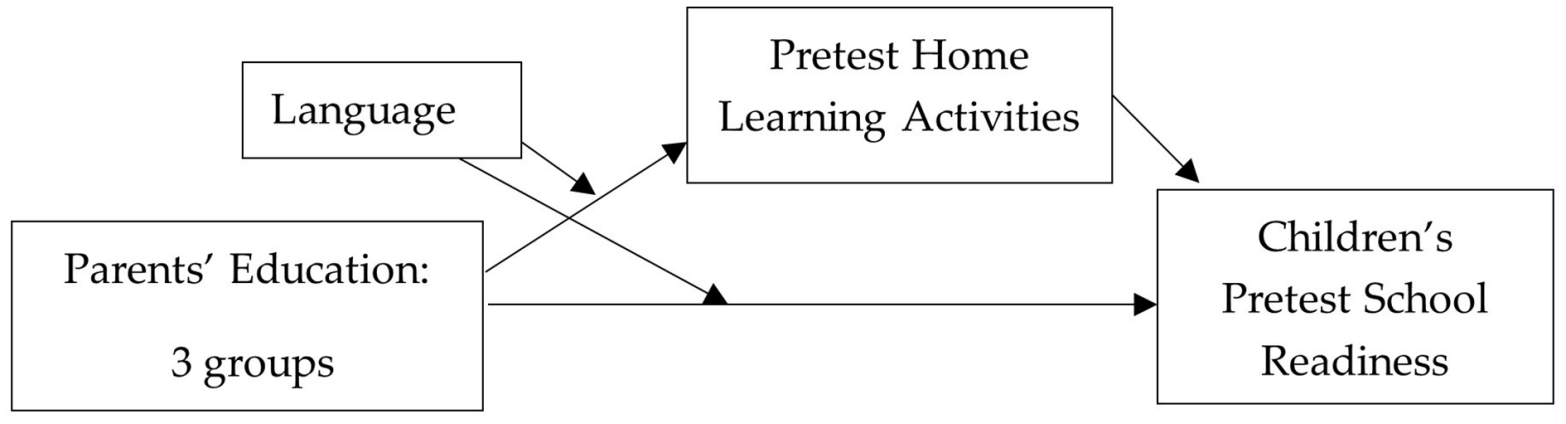
Proposed moderated mediation model of the relationship between parents’ education, language, home learning activities, and children’s school readiness scores.

Previous research has found that families’ participation in the HIPPY program increases parent involvement (e.g., Johnson et al., 2012) and children’s school readiness (e.g., Payne et al., 2020). However, limited research demonstrates how the structure of the program, which is built around role playing, modeling, and scaffolding, provides parents learning activities that engage children in these activities directly and contributes to a successful outcome such as school readiness. The current study examines the moderator and mediating effects of language and HLA, respectively, on the relation between parents’ education level and children’s pre-academic school readiness scores within the context of a home visiting program (Figure 1). The study also explores these relations before and after enrolment in the HIPPY home visiting program to assess how families perform at the beginning of the program and if the patterns change by the end of the program year. This knowledge will enhance our understanding of child development and the effectiveness of the HIPPY program and will enable home visiting interventions to better serve families.

The research questions addressed are:

- Do home learning activities mediate the association between parents’ level of education and children’s school readiness and does language moderate the direct and indirect effects of education before the HIPPY program (at pretest)?
- Do home learning activities mediate the association between parents’ level of education and children’s school readiness and does language moderate the direct and indirect effects of education at the end of the HIPPY program year (at posttest)?
- To what extent are these relations different at the beginning and end of the HIPPY program year?

We hypothesized that parents’ preferred language for program delivery (i.e., English or Spanish) would moderate the association between parent education and school readiness at pretest. However, we anticipated that increases home learning activities associated with program participation would mediate this relation between language preference and parent education at posttest alleviating the moderating effect of these characteristics on children’s school readiness. We expected different relations at posttest to indicate the program is serving families equitably.

## 2. Materials and methods

### 2.1. Participants

Participants were caregivers and their children who were enrolled in the HIPPY program in Texas. Families were recruited for the program across 11 program sites in Texas in communities where children faced a combination of risk factors (i.e., high poverty levels, social isolation, low parent education levels, lack of parent involvement, and lack of English proficiency) that may influence school readiness. Recruitment was conducted by home visitors who were members of the same community with most having been former HIPPY parents enrolled in the program. At enrollment, families could agree to their data being used for research and evaluative purposes. The study and request were approved by the authors University Institutional Review Board. There were 1,325 children who completed the pretest or posttest school readiness assessments, and 1,234 parents who completed the parent involvement assessments. However, only families that had completed the demographic survey and both assessments at pre- and post-test (N = 568 dyads) were included in the analyses. Of the 568 children and parents, 80% were Hispanic. Of the children, 51% were female. At pretest, children were between 36 and 75 months old (*M_age_* = 47.60 months, *SD* = 7.72) and between 42 and 82 months old (*M_age_* = 55 months, *SD* = 7.75) at posttest. Most participating adult family members were mothers (95.2%), but other program dyad participants were grandparents (2.3%), fathers (0.8%), legal guardians (0.8%), aunts (0.7%), and stepparents (0.2%). Parents’ highest level of education was categorized into three levels: less than high school (29%), high school (35%), and education beyond high school (36%). Families chose to receive the HIPPY program in Spanish (57%) or English (43%) based on their language preference.

### 2.2. Procedure

Data were obtained from families enrolled in HIPPY from 11 different sites across the State of Texas during the 2018–2019 program year. Following university IRB approval (IRB protocol 18–253), all HIPPY families provided informed consent during program enrollment, stipulating certain program evaluation data, including all data used for the present study, may be collected and used for research. At the beginning of the program year (August 2018), caregivers completed a demographic survey and the Parent Involvement Inventory (PII; adapted from Britto and Brooks-Gunn, 2003) and home visitors assessed children’s pre-academic skills using the Bracken School Readiness Assessment Third Edition (BSRA-3; Bracken, 2007). Across the 30-week program, home visitors went to the families’ homes once a week and spent 45–60 min introducing and role-playing activities to teach parents’ methods for implementing the curriculum materials with their children. The HIPPY curriculum includes activity packets, books, and materials for teaching math, language, literacy, science, and motor skills. Parents could opt to complete the program and assessments in English or Spanish. After each home visit, parents were encouraged to spend 15–20 min a day, 5 days a week, working on activity packets with their children. At the end of the program year (May 2019), the home visitor completed the BSRA-3 with the children and parents completed the PII in the same language they completed the program.

### 2.3. Measures

#### 2.3.1. Demographic information

Parents completed a survey with demographic questions when they enrolled in the program. Items included questions about the parents’ and child’s age, race, ethnicity, gender, and primary language, parents’ highest education level, and household size.

#### 2.3.2. Bracken school readiness assessment

The BSRA-3 is a normed measure that assess children’s performance on school readiness skills through the combination of five basic kindergarten academic categories: colors, letters, numbers, sizes, and shapes. The BSRA-3 takes approximately 15 min to complete and consists of 85 items in five sub-tests: colors (10 item), letters (15 items), numbers (18 items), sizes (22 items), and shapes (20 items). The School Readiness Composite (SRC), or raw test score, is calculated by adding the number of correct answers and can range from 0 to 85. SRC scores are converted to standard scores based on the age of the child at the time of assessment to compare the performance of children in the sample to the general population of children of the same age nationwide. Standard scores range from 40 to 160 with a mean of 100 and standard deviation of 15. Five descriptive classifications can be derived from school readiness standard scores: very delayed (40–70), delayed (71–85), average (86–114), advanced (115–129), and very advanced (130–160). Both the English and Spanish versions of the BSRA-3 are valid and reliable across multiple populations (Bracken, 2007; Ortiz et al., 2015). Psychometric properties of the BSRA-3 have demonstrated that children’s performance on the instrument predicts first grade readiness (Panter and Bracken, 2009) and children’s reading readiness at the end of kindergarten (Panter, 2000) The items in our sample had high internal reliability (Cronbach’s alpha = 0.84).

#### 2.3.3. Parent involvement inventory

The PII was used to assess the home learning activities (HLA) parents engaged in. Home visitors read the questions to parents in an interview format and recorded the parents’ answers. The PII takes approximately 15 min to complete and includes seven questions on the frequency of parents’ participation in educational and cognitively stimulating activities with their child. For instance, “How many times have you or someone in your family read to your child in the past week?,” “How often did you teach your child numbers?,” “How often did you do activities with your child that involve making patterns?,” and “How often did you do activities to help your child learn shapes?” The frequency with which parents engage in language, science, math, and spatial skills related activities with their children [0 = not at all, 1 = once or twice, 2 = 3–6 times, and 3 = every day] were examined in the current study. The 14 items included were the frequency of teaching the child letters; teaching words; teaching numbers; teaching shapes; making pattern activities; arranging objects by size, height, or color; doing counting activities; playing with toys for building (e.g., LEGO); playing board games, card games, and puzzles; playing games using dice or number pieces; talked about science or did a science project; reading books; singing songs; and telling stories. An HLA composite score was created for the 14 items and scores could range between 0 and 42. The items had high internal reliability (Cronbach’s alpha = 0.87).

### 2.4. Data analysis

Two conditional process models that combined simple mediation with moderation were conducted using the PROCESS macro version 4.0 (Hayes, 2022) in the Statistical Package for the Social Sciences (SPSS). Indirect effects were tested using a percentile bootstrap estimation approach with 5,000 samples, implemented with the PROCESS macro. For both models, the predictor variable was parents’ education level [levels: High = more than high school (coded 0); Intermediate = high school (coded 1); Low = less than high school (coded 2). The high education group was used as the reference group. The moderator was the language parents’ chose for program participation [0 = English, 1 = Spanish]. For the first model, the mediator was the frequency of HLA at pretest and the outcome variable was children’s pretest standardized school readiness scores. For the second model, the mediator was HLA at posttest and the outcome variable was children’s posttest standardized school readiness scores. Pretest school readiness was the covariate. Each model examined if there was an indirect effect of parents’ education on school readiness through HLA, and if language was changing the strength of the mediation. Additionally, each model also examined the moderating role of language on the direct effect of education on school readiness, that is, if the magnitude of the direct effect was dependent on language.

## 3. Results

### 3.1. Preliminary analyses

The composite score for HLA at pretest had an average of 21.96 (*SD* = 8.72, range = 0 to 45, *N* = 531) and 27.93 (*SD* = 7.27, range = 4 to 45, *N* = 555) at posttest. A paired samples t-test revealed that there was a statistically significant increase in HLA scores from pretest to posttest, *t*(499) = 15.48, *p* < 0.001, *d* = 0.69. Children’s pretest school readiness pretest scores ranged between 49 and 128 with an average of 85.05 (*SD* = 14.44), which is 1 standard deviation below the average. The descriptive classifications corresponding to children’s standard scores indicated that 15.8% of children were very delayed, 38.4% delayed, 41.1% average, and 4.5% advanced. At the posttest, standard school readiness ranged between 43 and 139 with an average of 96.71 (*SD* = 16.18). The descriptive classifications indicated that 5.2% of children were very delayed, 18.7% delayed, 62.2% average, 11.5% advanced, and 2.3% very advanced. Children’s school readiness scores also improved significantly from pretest to posttest, *t*(596) = 21.95, *p* < 0.001, *d* = 0.90. Correlations between the variables used in the models are shown on Table 1.

**Table 1:** Correlation matrix of variables used in the models.

	1	2	3	4
1. Pretest home learning activities score				
2. Pretest children’s school readiness score	0.30**			
3. Posttest home learning activities score	0.39**	0.08		
4. Posttest children’s school readiness score	0.30**	0.63**	0.15**	
5. Parents’ education level	−0.27**	−0.30**	−0.15**	−0.12**

**p < 0.001.

### 3.2. Model building

The conditional process models were built systematically. First, a linear regression was run to examine parent involvement predicted children’s school readiness scores. Parents’ education level significantly predicted children’s pretest school readiness scores, *F*(1,596) = 57.30, *p* < 0.001. Parents’ education level accounted for 8.8% of the explained variability in school readiness. For each change in education level (high to intermediate to low) school readiness scores decrease by 5.25 points. Next, language was included as a moderator between parents’ education and children’s school readiness. There was an interaction between parents’ education (high vs. intermediate) and language (*p* = 0.017). Then, HLA was included as a mediator between parents’ education and school readiness, without program language as a moderator. HLA mediated the relation between parents’ education level (high vs. intermediate) and children’s school readiness scores, (*B* = −1.61, *SE* = 0.46, 95% CI [−2.59, −0.81]) and between parents’ education (high vs. low) and school readiness (*B* = −2.13, *SE* = 0.52, 95% CI [−3.25, −1.21]). Finally, the full model was run including mediation and moderation.

### 3.3. Model 1: pretest school readiness

**Figure 2:**
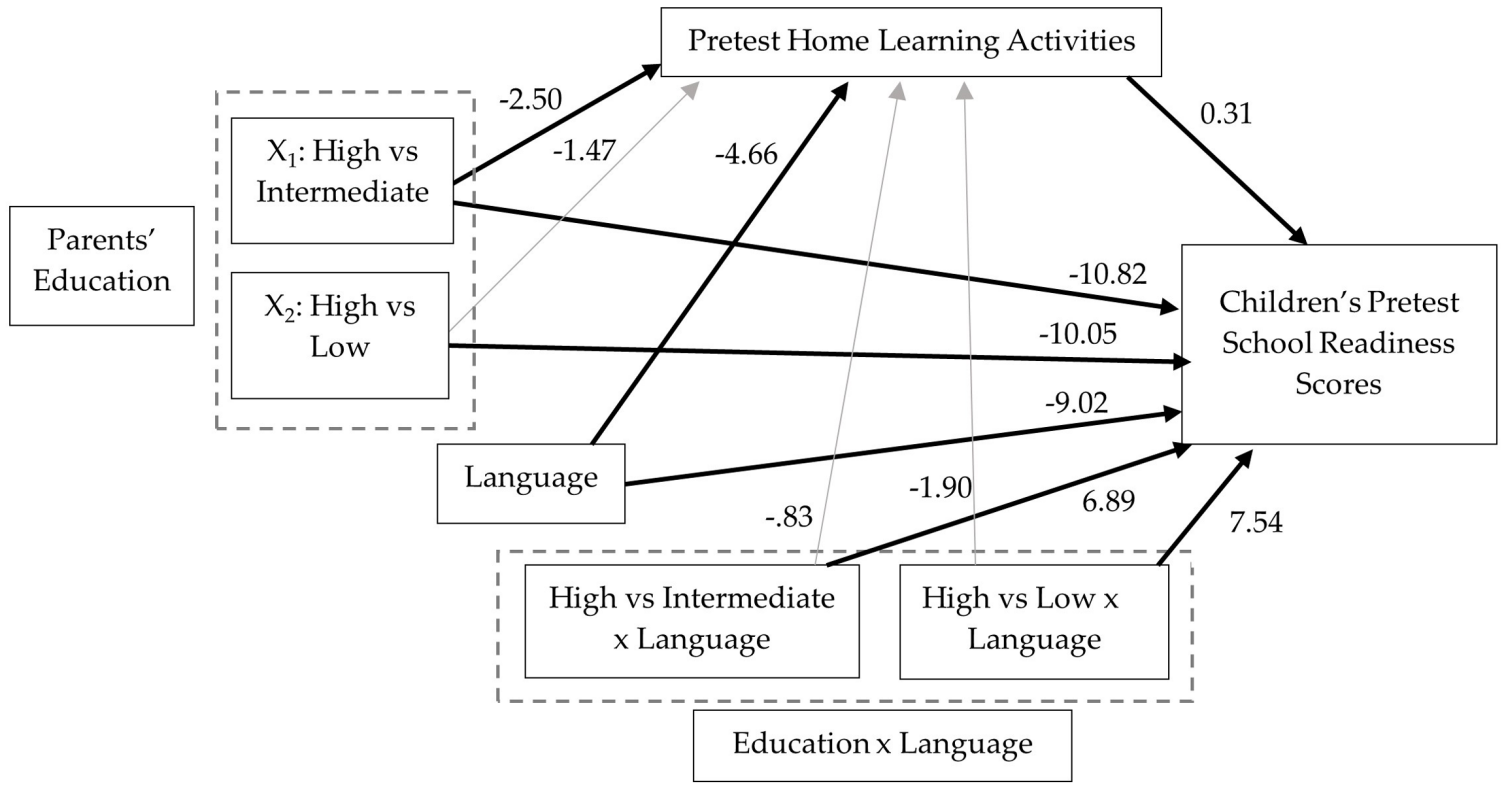
Path diagram of the relation between parents’ education, program language, and home learning activities on children’s school readiness scores at pretest. Solid dark arrows represent statistically significant paths.

Moderated mediation analysis was used to examine if parents’ involvement in HLA mediates the effect of parents’ education level on children’s school readiness scores at pretest and if the direct and indirect effects were moderated by program language. The path diagram with corresponding coefficients is shown in Figure 2.

**Figure 3:**
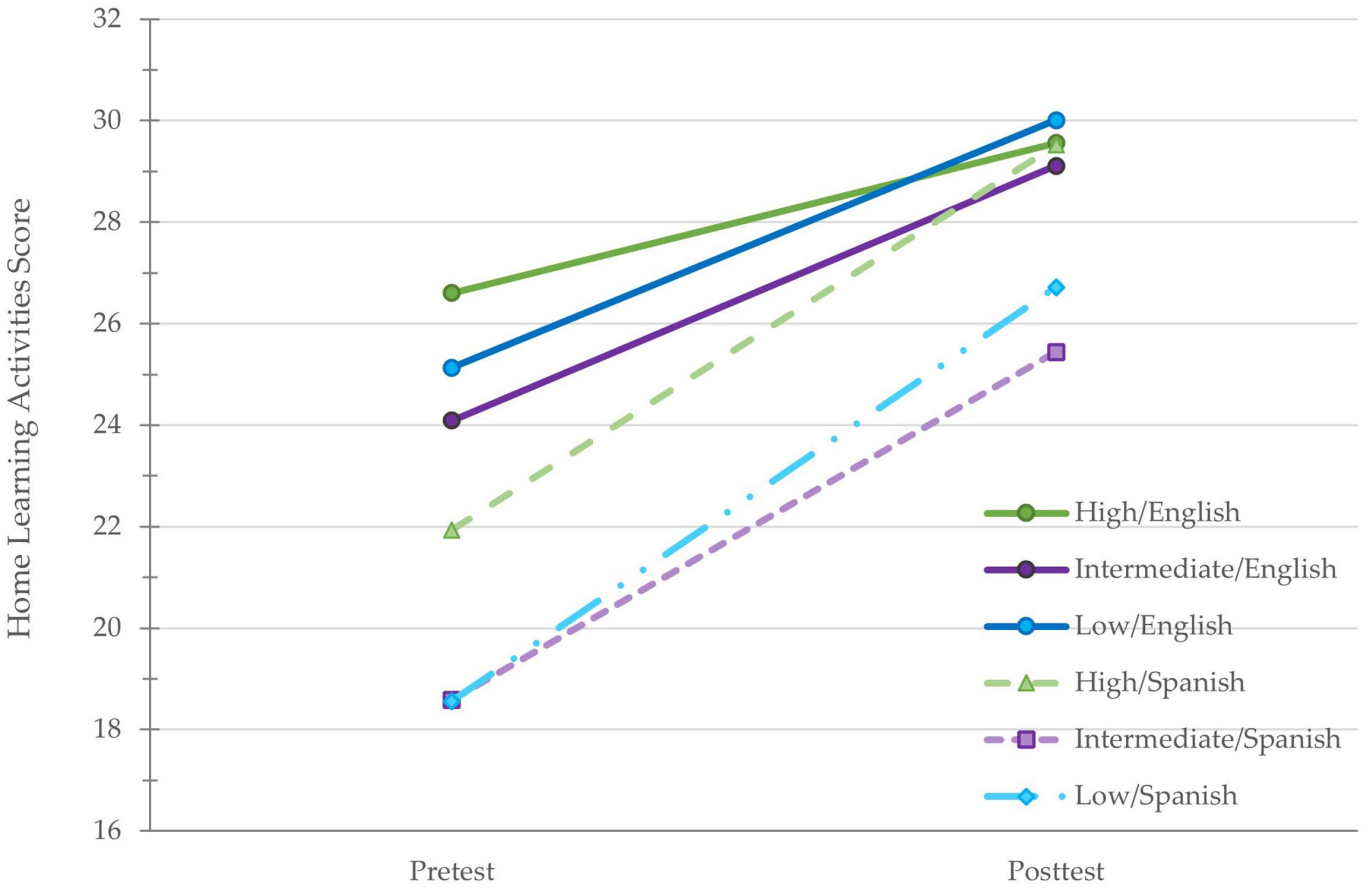
Mean home learning activities scores at pretest and posttest by education level and language.

Results indicate that the mean difference in HLA between families reporting intermediate and high levels of education was 2.50 ([*β* = −0.34], *SE* = 1.17, *t* = −2.14, *p =* 0.033) and between families reporting low and high levels of education was 1.47 ([*β* = −0.29], *SE* = 01.83, *t* = −0.80, *p* = 0.422). Program language had a significant effect on HLA (*B* = −4.66, [*β* = −0.27], *SE* = 1.27, *t* = −3.68_,_ *p* < 0.001). However, the interactions between parents’ education level (high vs. intermediate and high vs. low) and language were not statistically significant, *p* = 0.635 and *p* = 0.390, respectively (Figure 3). The effect of HLA on school readiness, controlling for parents’ education was statistically significant (*B* = 0.31, *[β* = 0.19], *SE* = 0.07, *t* = 4.44_,_ *p* < 0.001).

HLA mediated the relation between parents’ education and children’s school readiness scores for families completing the program in Spanish. The indirect effect of parents’ education (high compared to intermediate) on children’s school readiness through HLA was statistically significant, *B* = −1.04, (*β* = −0.07), *SE* = 0.51, 95% CI [−2.14, −0.17]. Compared to a family with high levels of education, a child from a family with intermediate levels of education completing the program in Spanish is estimated to differ by 1.04 points in their school readiness score as a result of more HLA for the high education group which in turn translates into higher school readiness scores. The indirect effect of parents’ education (high compared to low) on children’s school readiness through HLA was also statistically significant, *B* = −1.05, (*β* = −0.07), *SE* = 0.47, 95% CI [−2.11, −0.24]. For families completing the program in English, HLA mediated the relation between parents’ education (high compared to intermediate) and children’s school readiness scores, *B* = −0.78, (*β* = −0.05), *SE* = 0.40, 95% CI [−1.65, −0.08], but not for the high compared to low parents’ education group, *B* = −0.46, (*β* = −0.03), *SE* = 0.59, 95% CI [−1.69, 0.73].

The relation between language and school readiness was statistically significant (*B* = −9.02, [*β* = −0.31], *SE* = 2.07, *t* = −4.35, *p* < 0.001), which suggests that among families with high levels of education, children who received the program in English compared to Spanish are estimated to differ by 9.02 points on school readiness scores. Language moderated the effect of high education compared to intermediate education on children’s school readiness, (*B* = 6.89, [*β* = 0.25], *SE* = 2.83, *t* = 2.43, *p* = 0.015). The difference in school readiness scores between intermediate and high education changes by 6.89 points from English to Spanish. Language also moderates the effect of low education compared to high education on children’s school readiness, (*B* = 7.54, [*β* = 0.26], *SE* = 3.57, *t* = 2.43, *p* = 0.035). The difference between low and education is estimated to change by 7.54 points from English to Spanish.

**Figure 4:**
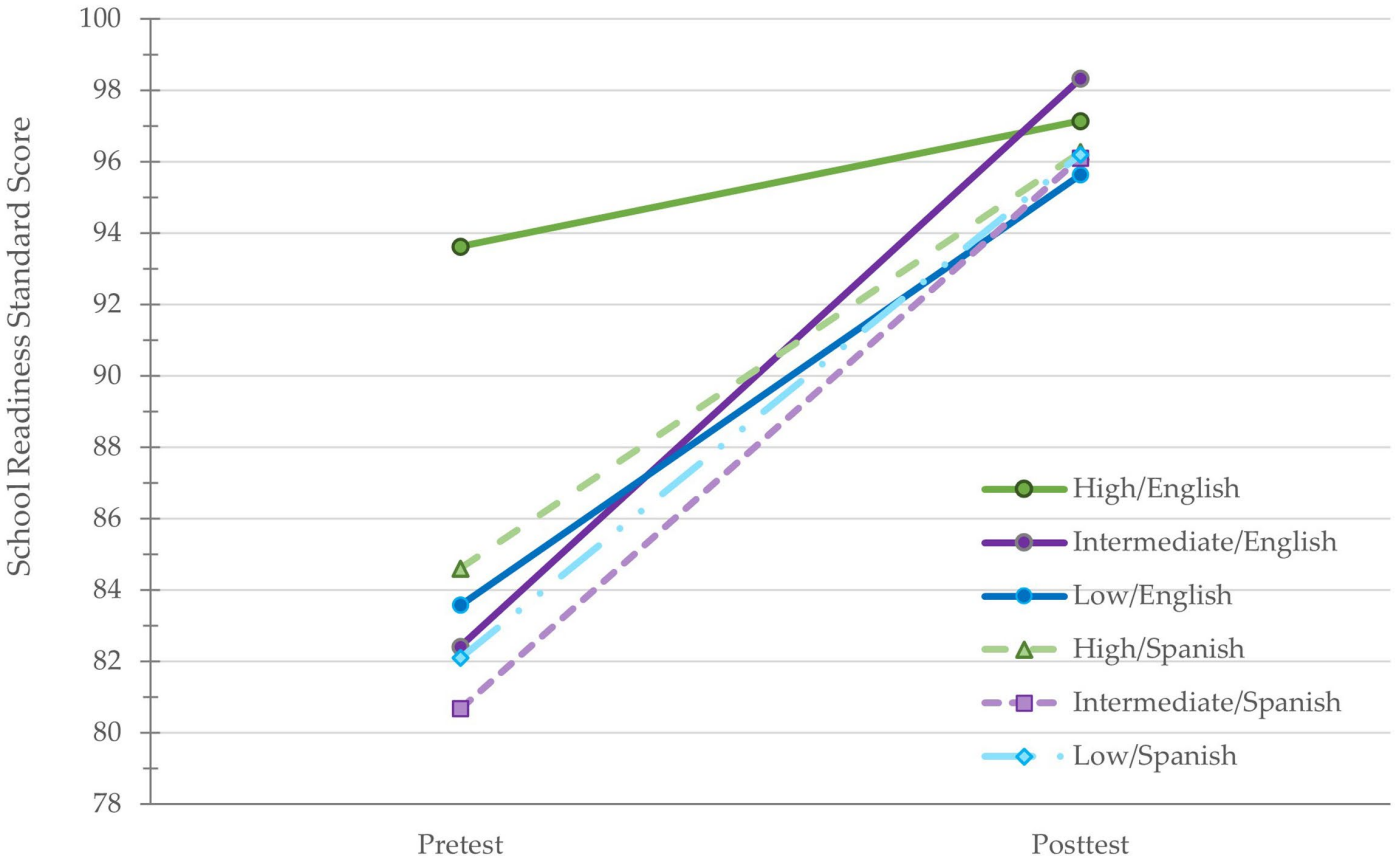
Program language was a significant moderator of the relation between parents’ education and children’s school readiness scores at pretest but not at posttest.

For families completing the program in English, children with parents in the intermediate education group are estimated to score 10.82 points lower in school readiness than children from families with high levels of education, see Figure 4. The relative conditional direct effect was statistically significant, (*B* = −10.82, [*β* = −0.78], *SE* = 1.90, *t* = −5.70, *p* < 0.001). Similarly, children with families in the low education group are estimated to score 10.05 points lower in school readiness than children in high education families. The relative conditional direct effect was statistically significant, (*B* = −10.05, [*β* = −0.70], *SE* = 2.95, *t* = −3.41, *p* = 0.001). For families completing the program in Spanish, the relative conditional direct effects were not statistically significant for families in the intermediate education group compared to the high education group (*B* = −3.93, [*β* = −0.27], *SE* = 2.13, *t* = −1.85, *p* = 0.065) or the low education group compared to the high education group (*B* = −2.50, [*β* = −0.17], *SE* = 2.04, *t* = −1.23, *p* = 0.219).

### 3.4. Model 2: posttest school readiness

**Figure 5:**
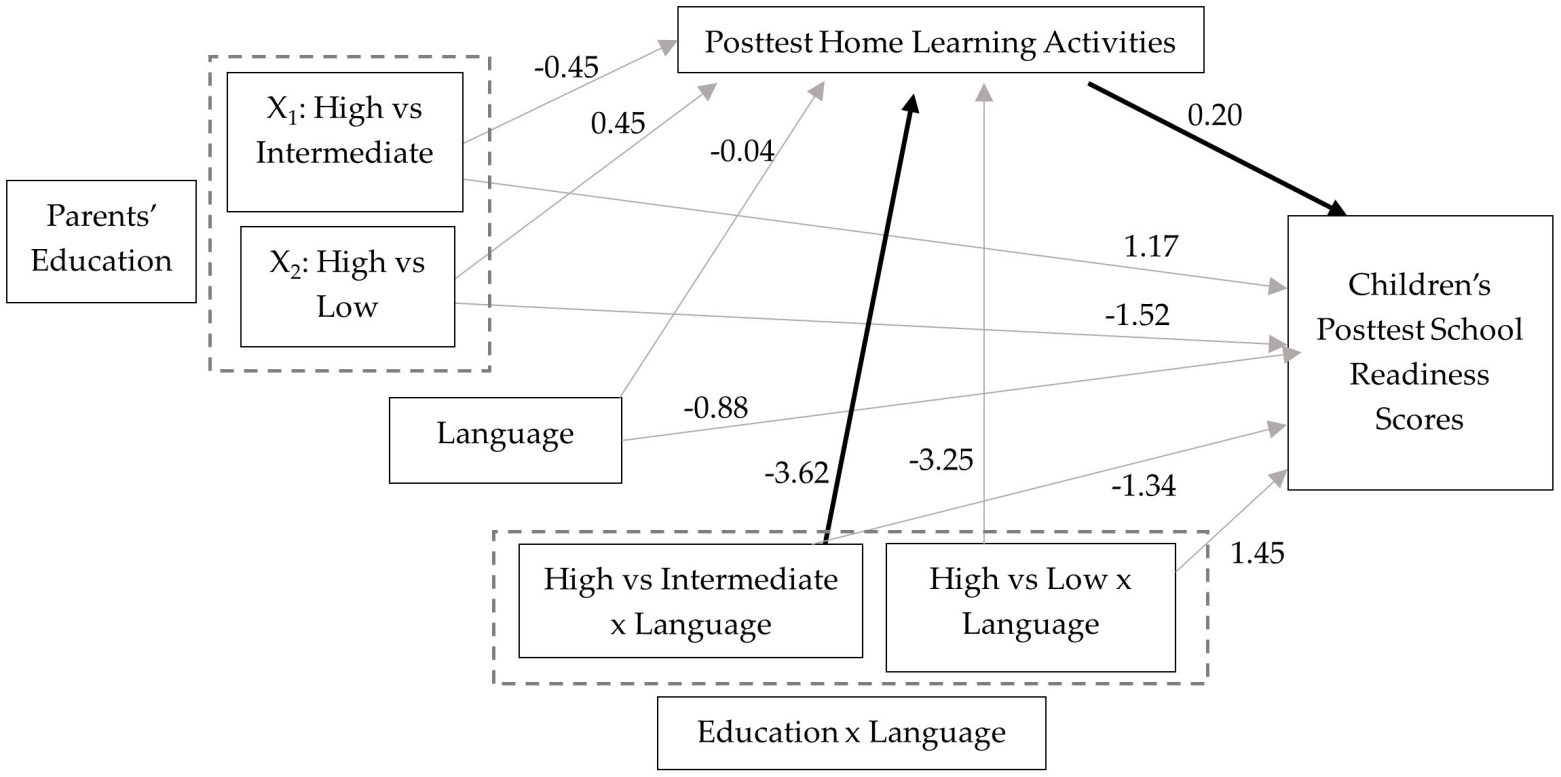
Path diagram of the relation between parents’ education, program language, and posttest home learning activities on children’s posttest school readiness scores. Dark arrows represent significant paths; light gray arrows represent non-significant paths.

Moderated mediation analysis was used to examine if HLA at posttest mediated the relation between parents’ education and children’s posttest scores in school readiness and if the effect was moderated by program language, controlling for pretest scores. Figure 5 presents the path diagram with unstandardized coefficients. The mean difference in HLA at posttest between families with intermediate and high levels of education (*p* = 0.664) and between families from low and high levels of education (*p* = 0.801) were not statistically significant. The interaction between parents’ education level (high vs. intermediate) and language was statistically significant (*B* = −3.63, [*β* = −0.25], *SE* = 1.52, *t* = −1.55, *p* = 0.017). The effect of HLA on school readiness posttest scores, controlling for parents’ education, was statistically significant (*B* = 0.20, [*β* = 0.10], *SE* = 0.08, *t* = 2.67, *p* = 0.001).

Children from families with intermediate levels of education receiving the program in English were estimated to score 1.17 points higher on school readiness than children from high education families, but this relative conditional direct effect was not statistically significant ([*β* = 0.03], *SE* = 1.81, *t* = 0.65, *p* = 0.517). Children from families with low education level receiving the program in English were estimated to score 1.52 points lower on school readiness than children from families with high education level, but the relative conditional effect was not statistically significant, ([*β* = −0.10], *SE* = 3.16, *t* = −0.48, *p* = 0.631).

The index of moderated mediation (−0.73) was statistically significant, *SE* = 0.42, 95% CI [−1.68, −0.06]. The indirect effect of parents’ education (high compared to intermediate) on children’s school readiness through HLA for families completing the program in Spanish was statistically significant, *B* = −0.81, (*β* = −0.05), *SE* = 0.38, 95% CI [−1.66, −0.17]. The indirect effect of parents’ education (high compared to low) on children’s school readiness through HLA was also statistically significant, *B* = −0.55, (*β* = −0.04), *SE* = 0.32, 95% CI [−1.30, −0.05]. For families completing the program in English, the indirect effects of parents’ education (high compared to intermediate and high compared to low) on children’s school readiness through HLA were not statistically significant.

Language did not moderate the direct effect of intermediate education compared to high education on children’s posttest school readiness scores (*B* = −1.34, [*β* = −0.02], *SE* = 2.68, *t* = −0.50 *p* = 0.618) or the effect of high education compared to low education on children’s school readiness (*B* = 1.45, [*β* = 0.05], *SE* = 3.68, *t* = 0.39, *p* = 0.695), Figure 4.

## 4. Discussion

The goal of the present study was to examine the relation between parents’ education level, program language, HLA, and school readiness before and after completing a year of the HIPPY program for a predominately Hispanic sample in Texas. At pretest, program language moderated the direct relation between parents’ education level and school readiness but did not moderate the indirect relation between education and school readiness through HLA. At posttest, language no longer moderated the association between parents’ education and school readiness but moderated the indirect relation between education and school readiness through parent involvement for families completing the program in Spanish. These findings provide a better understanding of the variables associated with school readiness for families enrolled in the HIPPY program.

Consistent with previous HIPPY evaluations, school readiness scores improved from pretest to posttest (e.g., Palladino, 2015). Based on descriptive classifications corresponding to school readiness scores, at pretest, 55% of children were delayed or very delayed but at posttest, only 24% were delayed or very delayed. Notably, unlike other studies and HIPPY evaluations that used teacher ratings to assess children’s school readiness (Johnson et al., 2012; Brown and Johnson, 2014), we used BSRA-3 standard scores that accounted for children’s age at assessment which provided a more robust measure of children’s performance and skills. HLA also improved from pretest to posttest, which was consistent with previous research on the effect of HIPPY on parents’ behaviors (Johnson et al., 2012; Brown and Johnson, 2014; Palladino, 2015).

At pretest, the direct effects of parents’ education level on school readiness were statistically significant and had large effects suggesting that a lot of the difference in school readiness scores could be attributed to education. Our findings are consistent with previous studies that have found associations between parents’ education level attainment and school readiness (e.g., West et al., 2000; Ghandour et al., 2021). Children’s scores improved regardless of their parents’ level of education. Notably, at posttest there the direct effects were no longer statistically significant, suggesting that the program is providing equitable support to families from different educational backgrounds.

Language moderated the relation between education and school readiness at pretest. The difference was particularly salient for the high education group completing the program in English, since their children had the highest school readiness scores. At posttest, however, language no longer moderated the relation between parents’ education and school readiness. Regardless of language, children of parents with different education attainment levels had similar posttest school readiness standard scores which suggests that HIPPY is serving families equitably in both languages. These findings also highlight why interventions and assessments should be conducted in families’ primary language. Assessing children in their primary language can help disentangle low academic skills and low English proficiency (López and Foster, 2021).

At pretest, parents with high levels of education were more likely to engage their children in educational activities than parents with intermediate levels of education. At posttest, however, there were no significant differences HLA based on parents’ highest education level. This information has practical implications because it suggests that intervention programs such as HIPPY that focus on supporting parents in developing their children’s pre-academic skills can encourage parents from different educational backgrounds to become more involved in HLA.

Furthermore, HLA was associated with children’s school readiness scores, regardless of parents’ highest level of education and language. This is consistent with previous research that has found a relation between HLA and school readiness (e.g., Fantuzzo et al., 2000; Evans, 2004) and supports the claim that promoting HLA by finding ways to get parents to engage more with their children is a strategy to improve children’s school readiness. Early interventions aimed toward enhancing parent engagement in learning activities can improve children’s cognitive skills, especially among low SES families (Marti et al., 2018). HIPPY home visitors emphasize that the skills and activities parents learn during home visits are not limited to the activity packets and the curriculum but can and should be applied during day-to-day activities without the need of elaborate materials. For instance, parents are encouraged to talk about shapes and colors when they are at the grocery store, cooking dinner, or driving. By noticing that they can use any opportunity to teach their children and engage them in conversations, parents become more involved in their child’s education and gain more confidence, self-efficacy, and embrace the idea that they are their children’s first teachers. These formal and informal learning interactions are likely helping children in their cognitive development.

Previous studies report that having a stimulating learning environment mediated the association between income and school readiness (e.g., Yeung et al., 2002). We extend these findings by using parents’ education level instead of income and by examining if language moderated the mediation both before and after enrollment in a HIPPY program year. At pretest, the Spanish group scored 4.66 points lower on HLA than the English group, but there was no interaction between education and language. At posttest, however, the indirect effect of parent education on school readiness through HLA operated to varying degrees depending on language. For the English group, language did not moderate the relation between education and HLA; that is, all education groups reported similar HLA. For the Spanish group, the high education group scored as high as the English groups, but the low and intermediate groups reported significantly lower HLA. Even though parent involvement of intermediate and low education families who completed the program in Spanish increased significantly from pretest to posttest, more effort should be made to support and encourage them to engage in more HLA during the program.

Notably, cultural and SES differences need to be considered to understand parents’ interactions with their children and to deliver a program that will help them benefit the most. Hispanic parents, particularly immigrants, are less likely to engage in HLA associated with developmental skills necessary to succeed in the US education system (Padilla and Ryan, 2020; López and Foster, 2021). For example, many Hispanic parents report that their parents did not read to them when they were growing up (Reese and Gallimore, 2000; Gillanders and Jiménez, 2004), which is likely why they do not include reading in children’s daily routines. Some Hispanic parents also believe that academic instruction should occur at school, so they do not instruct their children at home to not interfere with the teacher’s work (Reese and Gallimore, 2000).

On the other hand, more recent studies suggest that Hispanic mothers display resiliency and creativity to find ways to engage their children in home literacy activities even when they have limited English proficiency or books at home. Providing programs in Spanish for Hispanic families with Spanish-speaking home visitors may create a context in which the role-played parent–child interactions seem more culturally accepted. HIPPY shows parents that they are their children’s first teachers and gives them resources, training, and guidance to engage with their children at home in developmentally appropriate activities. The role-playing activities helps parents feel empowered and gain self-confidence and self-efficacy to teach their children (Nievar et al., 2011; Nathans et al., 2020) in the comfort of their home and with a peer from their community. HIPPY also provides developmentally appropriate resources and books in Spanish which is important because families report difficulty finding books in Spanish (Schick and Melzi, 2016; Avelar et al., in revision) but would read more if they had more books in Spanish (Coba-Rodriguez and Jarrett, 2022). When parents are encouraged to engage with their children in their native language, they can have more meaningful and language-rich conversations (Place and Hoff, 2011; Hammer et al., 2012), which in turn will help children’s cognitive development. However, providing materials, books, and activities to parents in a language in which they are not proficient, limits engagement in meaningful interactions.

### 4.1. Limitations and future directions

Because the current study used a pretest-posttest design and there was no randomization or control group, the findings do not explain causation and only reveal differences among families enrolled in HIPPY. Future studies should examine these relations to better understand the causal nature of the HIPPY program.

We operationally defined HLA as the frequency of engaging children in language and math related activities at home based on a self-report survey. As with any self-report measure, it is possible that parents may have reported the frequency of different activities inaccurately due to social desirability, difficulty recalling frequencies of each activity, or misunderstanding the questions. Furthermore, the current study only focused on frequency with which parents engage in activities with their children but examining the quality of these interactions is also important. Future studies should examine not only how frequently parents interact with their children but also *how* they interact, as quality may be playing a more important role in promoting school readiness skills than frequency. Future studies could also examine if there are certain behaviors or activities that have the largest impact on school readiness, or if it is a combination of different types of activities and engagement with children that drive this association. Finally, while this study focused only on parents’ or primary caregivers and how they interacted with their children, other family members could be engaging in learning activities with children, and this could also be affecting children’s outcomes. Thus, it is important to account for how HIPPY changes family interactions with children.

HIPPY focuses on helping parents to become more involved in educational activities with their children not only by completing the weekly activity packets, but also by participating in other activities outside the home. Parents attend group meetings with other parents in various places throughout the community to acquaint them with other HIPPY parents, which also serves to familiarize and establish comfort in visiting other places in their community. Families also attend field trips to libraries, museums, or zoos in their community where they are exposed to settings they would likely not visit on their own. Home visitors guide them and show them how to interact with their children with the hope that they will want to return with their family and provide rich learning activities for their children. Future research should investigate how home much involving parents in outside activities and with other parents contributes to parent involvement.

In this study, we focused on cognitive pre-academic skills that help children be ready for school entry using the BSRA-3. However, other measures such as teacher ratings could provide additional information of children’s school readiness. Given that behavioral and socioemotional skills are also key components of school readiness, future studies should also examine how they are associated with parents’ education level, language, and HLA. Children in this study were assessed in the language in which they completed the program. When children were assessed in Spanish, they performed comparably to English-speaking children in school readiness pre-academic skills. However, if Spanish-speaking children are assessed in English when they enter school, they may not have the necessary vocabulary to perform well in assessments even though they possess content knowledge. This has important implications and practical considerations for evaluations and avenues of future research, such as investigating how quickly children can transfer their knowledge from one language to another.

## 5. Conclusion

HLA plays a critical role in children’s school readiness. Even though school readiness is associated with parents’ education and language, providing parents the support they need early during development, helps parents interact with their children in fun learning activities, makes an important contribution preparing children for school, and, in turn, may have a long-term effect on academic achievement. Our findings highlight the vital role that the frequency of engagement in educational activities plays on children’s cognitive pre-academic skills. HIPPY is benefiting both English and Spanish speaking parents and children by helping parents to engage in more learning activities with their children. Even though the findings are for families enrolled in HIPPY, findings are also relevant for other programs, particularly those who work with similar populations as those enrolled in HIPPY. Programs such as HIPPY can work with parents to address the inequity of access by providing learning resources and training to promote student learning. The more relationships between family characteristics, HLA, and school readiness are understood, the better equipped families will be to support their children in school preparation.

## Data availability statement

The raw data supporting the conclusions of this article will be made available by the authors, without undue reservation.

## Ethics statement

The studies involving humans were approved by IRB protocol 18–253, University of North Texas. The studies were conducted in accordance with the local legislation and institutional requirements. Written informed consent for participation was not required from the participants or the participants’ legal guardians/next of kin in accordance with the national legislation and institutional requirements.

## Author contributions

DA, DH, and WM contributed to conception and design of the study. DA organized the dataset and performed the statistical analysis and wrote the first draft of the manuscript. DH and WM reviewed and edited the manuscript. All authors contributed to manuscript revision, read, and approved the submitted version.
